# Pre-clinical evaluation of quinoxaline-derived chalcones in tuberculosis

**DOI:** 10.1371/journal.pone.0202568

**Published:** 2018-08-16

**Authors:** Thaís C. Muradás, Bruno L. Abbadi, Anne D. Villela, Fernanda S. Macchi, Pedro F. Bergo, Talita F. de Freitas, Nathalia D. M. Sperotto, Luis F. S. M. Timmers, Osmar Norberto de Souza, Jaqueline N. Picada, Jean Fachini, Juliana Bondan da Silva, Nayara C. P. de Albuquerque, Maísa D. Habenschus, Daniel B. Carrão, Bruno A. Rocha, Fernando Barbosa Junior, Anderson R. M. de Oliveira, Alessandra Mascarello, Patrícia Neuenfeldf, Ricardo J. Nunes, Héctor R. Morbidoni, Maria M. Campos, Luiz A. Basso, Valnês S. Rodrigues-Junior

**Affiliations:** 1 Centro de Pesquisas em Biologia Molecular e Funcional (CPBMF) and Instituto Nacional de Ciência e Tecnologia em Tuberculose (INCT-TB), Pontifícia Universidade Católica do Rio Grande do Sul (PUCRS), Porto Alegre, RS, Brazil; 2 Programa de Pós-Graduação em Medicina e Ciências da Saúde, PUCRS, Porto Alegre, Brazil; 3 Programa de Pós-Graduação em Biologia Celular e Molecular, PUCRS, Porto Alegre, Brazil; 4 Laboratório de Bioinformática, Modelagem e Simulação de Biossistemas, PUCRS, Porto Alegre, Brazil; 5 Laboratory of Toxicological Genetics, Lutheran University of Brazil (ULBRA), Canoas, Brazil; 6 Departamento de Química, Faculdade de Filosofia, Ciências e Letras de Ribeirão Preto, Universidade de São Paulo, Ribeirão Preto, Brazil; 7 Laboratório de Toxicologia e Essencialidade de Metais, Faculdade de Ciências Farmacêuticas de Ribeirão Preto, Universidade de São Paulo, Ribeirão Preto, Brazil; 8 Departamento de Química, Universidade Federal de Santa Catarina (UFSC), Florianópolis, Brazil; 9 Laboratorio de Microbiología Molecular, Facultad de Ciencias Médicas, Universidad Nacional de Rosario (UNR), Rosario, Argentina; 10 Centro de Pesquisa de Toxicologia e Farmacologia, PUCRS, Porto Alegre, Brazil; University of Padova, Medical School, ITALY

## Abstract

New effective compounds for tuberculosis treatment are needed. This study evaluated the effects of a series of quinoxaline-derived chalcones against laboratorial strains and clinical isolates of *M*. *tuberculosis*. Six molecules, namely N5, N9, N10, N15, N16, and N23 inhibited the growth of the *M*. *tuberculosis* H37Rv laboratorial strain. The three compounds (N9, N15 and N23) with the lowest MIC values were further tested against clinical isolates and laboratory strains with mutations in *katG* or *inhA* genes. From these data, N9 was selected as the lead compound for further investigation. Importantly, this chalcone displayed a synergistic effect when combined with moxifloxacin. Noteworthy, the anti-tubercular effects of N9 did not rely on inhibition of mycolic acids synthesis, circumventing important mechanisms of resistance. Interactions with cytochrome P450 isoforms and toxic effects were assessed *in silico* and *in vitro*. The chalcone N9 was not predicted to elicit any mutagenic, genotoxic, irritant, or reproductive effects, according to *in silico* analysis. Additionally, N9 did not cause mutagenicity or genotoxicity, as revealed by *Salmonella*/microsome and alkaline comet assays, respectively. Moreover, N9 did not inhibit the cytochrome P450 isoforms CYP3A4/5, CYP2C9, and CYP2C19. N9 can be considered a potential lead molecule for development of a new anti-tubercular therapeutic agent.

## Introduction

Tuberculosis (TB), an infectious disease caused by *Mycobacterium tuberculosis*, was diagnosed in more than 10-million people in 2016, and is responsible for 1.45 million deaths per year. Patients diagnosed with active TB should initiate treatment immediately, completing the therapeutic scheme. However, adherence to inappropriate treatment regimens lead to development and spread of drug-resistant *M*. *tuberculosis* strains, which require more toxic, less effective and expensive drugs, and a longer therapeutic protocol [1,2].

Chalcones are compounds belonging to the flavonoid family, characterized by the presence of a chemical group 1,3-diaryl-2-propen-1-one. Their simple structure, the facility of substitution of hydrogens, combined with the ability for direct synthesis, facilitate structure-activity studies and target exploration [3–5]. Several pharmacological activities have been demonstrated for chalcones, including anti-tubercular activity [3,6–8]. In this regard, a series of chalcones displayed inhibitory effects on the growth of the *M*. *tuberculosis* H37Rv laboratorial strain [9].

Recent studies showed that quinoxaline derivatives also show anti-tubercular activity [10]. According to similarity of the chemical structure between some anti-TB drugs and the quinoxaline ring, as well as the presence of the quinoxaline fraction in some wide spectrum antibiotics, it is expected that quinoxaline analogues exhibit anti-tubercular activity. High potency, high selectivity index and low cytotoxicity have been observed after treatment with quinoxaline derivatives [11].

It has been suggested that chalcones and quinoxalines might be combined to potentiate their biological activities. For instance, a series of quinoxaline-derived chalcones presented marked anti-proliferative effects when tested in glioblastoma and oral cancer cells [12,13]. This work evaluated the efficacy and safety of a series of quinoxaline-derived chalcones as candidates for TB treatment.

## Materials and methods

### *Mycobacterium tuberculosis* susceptibility assays

#### Drugs and bacteria

**Fig 1:**
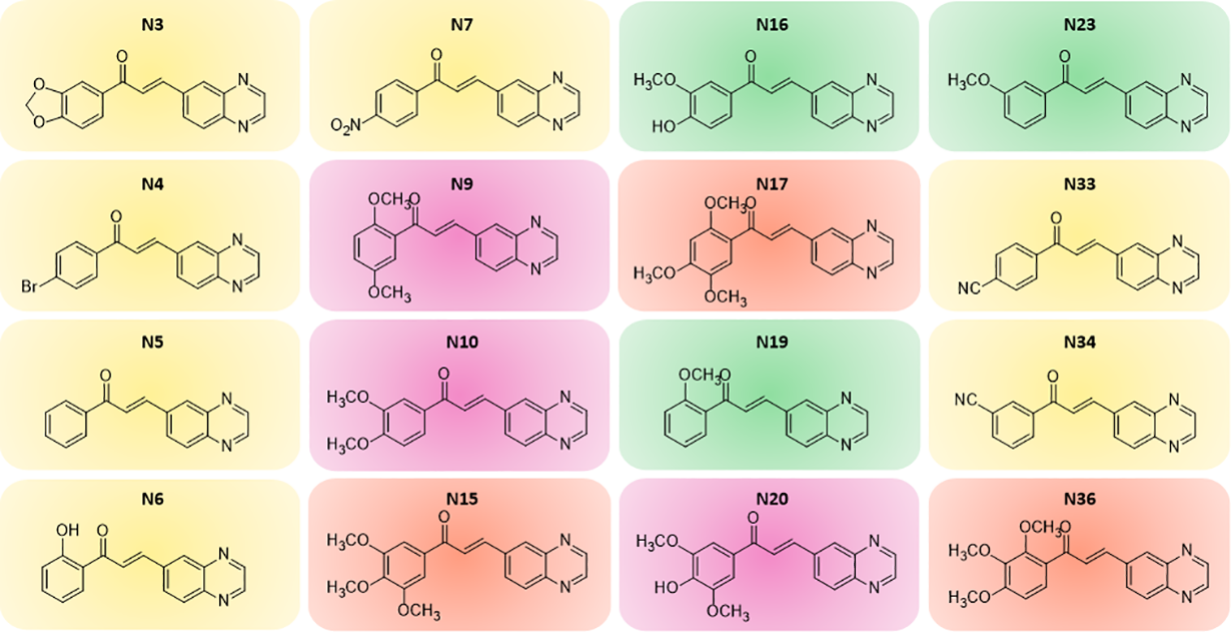
Chemical structures of the chalcones evaluated against the laboratorial *M*. *tuberculosis* H37Rv strain. The non-methoxylated structures are highlighted in yellow, the monomethoxylated molecules are in green, the di-methoxylated in pink, and the structures containing tri-methoxy radicals on the A-ring are in orange.

The series of quinoxalinic-derived chalcones (**Fig 1**) was synthesized and characterized essentially according to Mielcke et al. (2017) [13]. Rifampicin (RIF), ethambutol (EMB), and moxifloxacin (MFX) were purchased from Sigma-Aldrich, and isoniazid (INH) was purchased from ACROS Organics. *M*. *tuberculosis* strains were cultured as previously described [14].

#### Determination of minimum inhibitory concentrations (MICs)

Individual MICs for each tested compound were determined in *M*. *tuberculosis* H37Rv strain as previously reported [15]. Briefly, the compounds were first solubilized in DMSO at a concentration of 4 mg/mL, except for compounds N3 and N7 (1 mg/mL), and then diluted in Middlebrook 7H9 broth to reach a concentration of 50 μg/mL, or 10 and 20 μg/mL for compounds N6 and N23, respectively. Serial two-fold dilutions were performed in 96-well U-bottom polystyrene microplates at concentration ranges of 25–0.05 μg/mL for all compounds, except for N6 (5–0.01 μg/mL) and N23 (10–0.02 μg/mL). Mycobacterial suspensions were thawed and diluted in 7H9 medium at a theoretic optical density (OD_600nm_) of 0.006, and 100 μL were added to each well. Following 7 days of incubation at 37°C, 60 μL of a resazurin solution (0.01%) were added to the plates and the results were evaluated after 48 hours. MICs were determined by using the resazurin reduction microplate assay (REMA) as a growth indicator, and were considered as the lowest drug concentration that prevented a color change from blue (resazurin) to pink (resorufin) [16]. The values reported here were observed in two independent experiments, or were the highest value found among three independent tests. INH was used as a positive control drug. Compounds N9, N15 and N23 were subsequently tested against several resistant strains of *M*. *tuberculosis*: three drug-resistant clinical isolates (CDCT-10, CDCT-16, CDCT-27), one pan-susceptible strain (CDCT-28) and two laboratorial INH-resistant strains [pNIP::InhA(S94A) and pNIP::KatG(S315T)] (**Table 1**). All three clinical isolates carry the most common mutation found in the *katG* (*Rv1908c*) gene (S315T), CDCT-10 and CDCT-16 hold an additional mutation in the *rpoB* gene (H526D and D516V, respectively), responsible for causing resistance to RIF, and CDCT-16 also carries a mutation [C(-15)T] in the promoter sequence of the *inhA* (*Rv1484*) gene [17]. The pNIP::InhA(S94A) strain holds an additional copy of the *inhA* gene with a mutation that changed the naturally occurring Ser94 residue to an alanine, while the pNIP::KatG(S315T) strain had its wild *katG* gene knocked out and received a mutated copy of this gene, which changed the Ser315 to a threonine residue.

**Table 1:** Activity of chalcones against *M*. *tuberculosis* clinical isolates and laboratorial strains.

Compounds	MIC (μg/mL)^a^									Reference
	H37Rv	CDCT 10^b^	CDCT 16^b^	CDCT 27^b^	CDCT 28^b^	pNIP:: control^c^	pNIP:: InhA(S94A)^c^	pNIP:: KatG(WT)^c^	pNIP:: KatG(S315T)^c^	
INH	0.39	6.25	>100	25	0.39	<0.2	12.5	<0.2	>100	This study
RIF	<0.2	>100	>100	<0.2	<0.2					Abbadi, 2018
MFX	<0.2	<0.2	<0.2	<0.2	<0.2					Abbadi, 2018
N3	>25									This study
N4	>25									
N5	12.5									
N6	>5									
N7	>25									
N9	3.13	1.56	3.13	3.13	1.56	3.13	3.13	3.13	1.56	
N10	12.5									
N15	6.25	3.13	6.25	6.25	3.13	3.13	3.13	3.13	3.13	
N16	12.5									
N17	>25									
N19	>25									
N20	>25									
N23	5	2.5	5	5	2.5	2.5	2.5	2.5	1.25	
N33	>25									
N34	>25									
N36	25									

^*a*^MIC values reported here were observed in two independent experiments or the highest value found among three independent tests. INH, isoniazid; RIF, rifampicin; MFX, moxifloxacin.

^*b*^Drug-resistant (CDCT-10, CDCT-16, CDCT-27) and pan-susceptible (CDCT-28) clinical isolates: CDCT-10 holds mutations in *katG* (S315T) and *rpoB* (H526D) genes, CDCT-16 carries mutations in *katG* (S315T), *rpoB* (D516V), and in the promoter sequence of *inhA*, [C(-15)T], CDCT-27 holds mutation in *katG* (S315T), while CDCT-28 carries mutation in *rpoB* (D516A) gene.

^*c*^These strains were complemented with a wild-type [pNIP::KatG(WT)] or a mutant [pNIP::KatG(S315T)] copy of *katG* gene, or a mutant (S94A) copy of the *inhA* gene [pNIP::InhA(S94A)], or with empty pNIP vector [pNIP::control]. pNIP::InhA(S94A) and pNIP::KatG(S315T) are INH-resistant strains.

#### Combination of compound N9 with clinically used anti-TB drugs

The effects of the combination of N9 with the clinically used anti-TB drugs INH, RIF, EMB, and MFX was assessed by a checkerboard assay in a two-drug association scheme, together with the REMA colorimetric method as a growth indicator, as previously reported [15]. Briefly, all compounds were solubilized in DMSO at concentrations of 1 mg/mL (INH, RIF, EMB, MFX) or 4 mg/mL (N9). Then, the compounds were diluted in 7H9 broth to obtain a concentration range in microplates of 10–0.039 μg/mL for INH and EMB; 0.3–0.001 μg/mL for RIF and MFX; and 6.25–0.098 μg/mL for compound N9. Anti-TB drugs were diluted horizontally (columns 2 to 10), while the N9 compound was diluted vertically (rows B to H). The mycobacteria inoculum, the incubation conditions of the microplates and the readout of results were carried out as described before for MICs determination. Values of fractional inhibitory concentration index (FICI) below 0.5 indicate a synergism between the compounds, in between 0.5 and 4 indicate an indifferent influence, and above 4 suggest an antagonistic effect [15]. Three independent experiments were performed.

### *In silico* predictions

All compounds were submitted to the pkCSM webserver [18] to analyze their likelihood to interact with cytochrome P450 (CYP450), either as inhibitors, substrates, or as both. This analysis encompassed five isoforms of CYP450 (CYP1A2, CYP2C19, CYP2C9, CYP2D6, and CYP3A4). Compounds that act as inhibitors of CYP450 are contraindicated since they can affect drug metabolism. Moreover, molecules that serve as substrates to CYP450 can have their mode of action dramatically altered. Therefore, this analysis can be useful to predict adverse effects related to the CYP450 system. In addition, we also performed analysis for prediction of toxic effects (tumorigenic, mutagenic, irritant, and reproductive effects) for all compounds, using the DataWarrior program [19].

### CYP450 inhibition study

#### Chemical and reagents

The probe substrates phenacetin, acetaminophen, diclofenac, chlorzoxazone and nifedipine were purchased from Sigma-Aldrich (St. Louis, MO, USA); its metabolites 4′-hydroxydiclofenac, S-mephenytoin, 4′-hydroxymephenytoin, bufuralol, 1′-hydroxybufuralol, 6-hydroxychlorzoxazone and dehydronifedipine were acquired from Toronto Research Chemicals (Toronto, Canada); midazolam and 1′-hydroxymidazolam were obtained from Cayman Chemicals (Ann Arbor, MI, USA). Diazepam and tolbutamide, used as internal standards (IS), were purchased from Sigma-Aldrich (St. Louis, MO, USA). The following solutions were prepared: 962 μmol L^-1^ phenacetin, 8000 μmol L^-1^ S-mephenytoin, 432 μmol L^-1^ bufuralol, 11672 μmol L^-1^ chlorzoxazone, 560 μmol L^-1^ nifedipine, 433 μmol L^-1^ midazolam and 3956 μmol L^-1^ diclofenac. Except for diclofenac, which was prepared in methanol, all dilutions were made in acetonitrile. The IS diazepam and tolbutamide were prepared at a concentration of 0.2 μmol L^-1^ in methanol. All the solutions were stored in amber tubes at −20°C.

Human liver microsomes (HLMs), pooled from 150 donors, were purchased from Corning Life Science (Phoenix, AZ, USA). The constituents of the NADPH regeneration system, D-glucose-6-phosphate sodium salt, glucose-6-phosphate dehydrogenase, and β-nicotinamide adenine dinucleotide phosphate hydrate (NADP^+^), and macrogol glycerol ricinoleate (Cremophor EL) were acquired from Sigma-Aldrich (St. Louis, MO, USA). The solutions of the NADPH regeneration system were prepared in Tris-KCl buffer pH 7.4 (tris(hydroxymethyl)aminomethane 0.05 mol L^-1^ and KCl 0.15 mol L^-1^): 50 mmol L^-1^ D-glucose-6-phosphate, 8.0 U mL^-1^ glucose-6-phosphate dehydrogenase and 2.5 mmol L^-1^ β-nicotinamide adenine dinucleotide phosphate hydrate. These solutions were stored at −20°C.

HPLC grade solvents, such as methanol, ethyl acetate and chloroform were purchased from Panreac (Castellar Del Vallès, Spain). Analytical grade reagents, such as hydrochloric acid, sodium phosphate monobasic and sodium phosphate dibasic were acquired from Synth (Diadema, Brazil), while potassium chloride was obtained from Mallinckrodt Baker (Xalostoc, Mexico), and tris(hydroxymethyl)aminomethane from JT Baker (Phillipsburg, NJ, USA).

#### Analysis conditions

The analysis was performed using a Thermo Scientific LC system equipped with a pump (Accela 600 pump) and an autosampler coupled with Thermo Scientific TSQ Quantum Access Max and an electrospray triple quadrupole mass spectrometer. The chromatographic separation was carried out on an Ascentis Express Fused Core C18 column (100 x 4.6 mm ID, 2.7 μm particle size) connected to an Ascentis Express C18 guard column (3.0 mm x 4.6 mm ID, 2.7 μm particle size) purchased from Supelco (Bellefonte, PA, USA). The mobile phase comprised methanol: ultrapure water (90:10, v/v) that contained 0.1% of formic acid (v/v) for analysis of probes 1A2, 2C9, 2D6, 2E1 and 3A4/5, or 0.1% of ammonium hydroxide (v/v) for analysis of the CYP2C19 probe. Xcalibur software 2.0 (Thermo Fisher Scientific, Waltham, MA, USA) was used to control the instrument and to process data.

To optimize working conditions and to carry out the tandem mass spectrometry experiments, stock standard MeOH: ultrapure water (1:1, v/v) solution of each compound at concentration of 1 μg mL^-1^ was infused at a flow-rate of 50 μL min^-1^ using direct infusion via a syringe pump integrated into the TSQ instrument to mix the standard solution with the mobile phase (450 μL min^-1^; MeOH:H_2_O). Two selective reaction monitoring (SRM) transitions were used for each compound, one for quantification and one for confirmation (**S1 Table**).

Mass spectrometer conditions, such as cone voltage, capillary temperature, vaporizer temperature, sheath gas, ion sweep gas, and auxiliary gas, are summarized at **S2 Table**. Nitrogen was used as a sheath gas, ion sweep gas and auxiliary gas, and argon was used as a collision-induced-dissociation gas at 1.8 mTorr.

#### CYP450 inhibition experiments

To evaluate the potential of N9 to inhibit CYP450 enzymes, human liver microsomes were used to assess the rate of metabolism of the probe substrate of each CYP450 isoform, evaluated in the absence or the presence of N9. The concentration of N9 that caused 50% reduction of the activity of each CYP450 isoform (IC_50_) was determined. The enzyme inhibition assays were performed using a probe substrate for each CYP450 isoform. The probe substrate concentrations are equal to their K_M_ values, as summarized in the **S3 Table**. The compound N9 was evaluated at seven different concentrations: 10.0 nmol L^-1^, 46.4 nmol L^-1^, 215.4 nmol L^-1^, 1.0 μmol L^-1^, 4.6 μmol L^-1^, 21.5 μmol L^-1^, and 100 μmol L^-1^. Control samples were also tested in the absence of N9. The experiments were performed in triplicate.

The microsomal medium was composed by N9 solution (5 μL), probe substrate solution (5 μL), HLMs (90 μL), NADPH regeneration system solution (100 μL), and sodium phosphate buffer pH 7.4 0.1 mol L^-1^+ 0.34% (m/v) Cremophor EL (200 μL). The incubation conditions were previously determined by Habenschus et al. (2017) [20], and are described in **S3 Table**. After 5 min prewarmed at 37°C in a Dubnoff metabolic shaking incubator SL157 (Solab, Piracicaba, Brazil), the reactions were initiated by the addition of 100 μL of NADPH regeneration system solution. After the incubation period, the reaction was stopped by adding the extraction solvent (1.0 mL) and modifier, when necessary (**S3 Table**). The IS solution was then added (50 μL), and the samples were shaken in a Vibrax VXR agitator system (IKA. Staufen, Germany) for 15 min at 1000 rpm, and then centrifuged at 4°C and 1800 × g for 10 min (Hitachi CF16RXII, Tokyo, Japan). The extractor phase was collected (600 μL) and dried using a Concentrator Plus Speed Vacuum (Eppendorf, Hamburg. Germany). Then, the residue from each sample was solubilized in the mobile phase (200 μL) and analyzed by LC-MS/MS under the conditions described above. IC_50_ values were determined by a nonlinear regression of the percentage of remaining enzyme activity versus the logarithm concentration of N9. The GraphPad Prism Software 5.0 (San Diego, CA, USA) was used to analyze these results.

### Toxicity assays

#### *Salmonella*/microsome mutagenicity test

*Salmonella typhimurium* strains TA98, TA97a, TA100, TA1535, and TA102 were provided by MOLTOX^®^ (Molecular Toxicology Inc. USA). Mutagenicity was assayed according to the pre-incubation procedure following the recommendation described in Mortelmans and Zieger (2000) [21]. The S9 metabolic activation mixture (S9 mix) was prepared according to Maron and Ames (1983) [22]. Chalcone N9 was tested with the highest concentration limited by its solubility in DMSO, reaching 400 μg/plate. Briefly, 100 μL of test bacterial cultures (1–2 x 10^9^ cells/mL) were incubated at 37 ^o^C with different concentrations of N9 (10–400 μg/plate) in the presence or absence of S9 mix, for 20 min, without shaking. Subsequently, 2 mL of soft agar (agar 0.6%, NaCl 0.5%, histidine 50 μM, biotin 50 μM, pH 7.4, 42 ^o^C) were added to the test tube and poured immediately onto a plate of minimal agar (1.5% agar, Vogel-Bonner E medium, containing glucose 2%). 2-aminoanthracene (2-AA, 5 μg/plate) was used as positive control for all strains in the presence of metabolic activation (with S9 mix). In the absence of metabolic activation, 4-nitroquinoline-oxide (4-NQO, 0.5 μg/plate) was used for TA98, TA97a, and TA102 strains, and sodium azide (NaN_3_, 1 μg/plate) was used for TA100 and TA1535 strains. Plates were incubated in the dark at 37 ^o^C for 48 h before counting the revertant colonies. Assays were repeated twice and the plating for each concentration was conducted in triplicate. Data were evaluated by one-way analysis of variance (ANOVA), followed by Dunnett's post-test, using GraphPad Prism 5.0 (GraphPad, San Diego, CA, USA). Differences were considered significant at the 95% level of confidence.

#### Genotoxicity investigation

The alkaline comet assay was performed to evaluate possible genotoxic effects of N9. Human hepatocellular carcinoma (HepG2) cells were plated in a 6-well culture microtiter plate (5 x 10^5^ cells per well) in DMEM and allowed to adhere overnight prior to incubation for 24 h with N9 (2.5, 5, or 10 μg/mL). Methyl methanesulfonate (MMS; Sigma-Aldrich), a known mutagenic alkylating agent, was incubated for 1 h (100 μM), as positive control [23]. N9 solutions were prepared in DMSO, and the final concentration of DMSO in the assays was 0.5%. Sixty μL of HepG2 cell suspension was mixed with 180 μL of low-melting point agarose (Invitrogen) and scattered on two pre-coated microscope slides with normal agarose (Invitrogen). Cells were then lysed in freshly prepared ice-cold solution (10 mM Tris, 2.5 M NaCl, 100 mM EDTA with 10% DMSO and 1% Triton X-100), for 24 h. The slides were then incubated for 20 min at 4°C with fresh buffer (300 mM NaOH e 1 mM EDTA) to induce DNA unwinding and to expose the alkali-labile sites, with light protection. Electrophoresis was conducted for 20 min at 25 V and 300 mA. Slides were fixed and silver stained after neutralization. One hundred cells were scored according to the size and amount of DNA in the tail. Individually, the cells were given an arbitrary value of 0 (undamaged) to 4 (maximally damage) [24]. The statistical analysis was performed as described above for *Salmonella* mutagenicity test.

## Results and discussion

### Chalcones are active against drug-resistant clinical isolates and laboratory strains of *M*. *tuberculosis*, which are resistant to isoniazid

As shown in **Table 1**, six out of 16 chalcones (N5, N9, N10, N15, N16, and N23) inhibited the growth of the *M*. *tuberculosis* H37Rv laboratory strain, with MIC values ranging from 3.13 to 12.5 μg/mL. Three compounds (N9, N15 and N23) showed the lowest MIC values (3.13, 6.25, and 5 μg/mL, respectively) and, therefore they were further tested against four clinical isolates. None of the clinical isolates presented resistance to these compounds (**Table 1**), suggesting that the chalcones are capable of evading the major mechanisms of resistance found in clinical isolates of *M*. *tuberculosis*, such as mutations in either *katG* or *rpoB* genes. In addition, the compounds were also active against two laboratory strains holding mutations in the *katG* [pNIP::KatG(S315T)] or *inhA* [pNIP::InhA(S94A)] genes (**Table 1**), showing that the mechanism of action of the chalcones diverges from that observed for INH. This finding was further confirmed when we analyzed the effect on mycolic acid synthesis by *in vivo* radiolabeling extraction and analysis of lipids of *M*. *tuberculosis* cells after treatment with N9. These experiments revealed that N9 did not inhibit the synthesis of both mycolic acids and non-hydroxylated fatty acids, behaving differently from INH (**S1 Fig**). In this regard, a series of chalcones was recently suggested to be InhA inhibitors, by molecular docking analysis. However, the potential of these molecules to abrogate mycolic acids synthesis was not evaluated [10].

### The chalcone N9 displays a synergistic effect in combination with MFX

In order to evaluate the activity of the chalcones in combination with other anti-TB drugs (INH, RIF, EMB and MFX), a checkerboard assay was performed in *M*. *tuberculosis* with the lead compound N9. As shown in **Table 2**, this compound showed an indifferent effect when combined with INH, RIF and EMB (FICI values between 0.5 and 4.0). On the other hand, the compound N9 displayed a synergistic effect in the presence of MFX (FICI values below 0.5 in three independent experiments). The MIC of N9 in the presence of MFX was improved 8-fold in two experiments, and 32-fold in the other, while the MIC of MFX in the presence of N9 was improved 4-fold in all three experiments (**Table 2**). No combination presented antagonistic interactions in our experiments.

**Table 2:** Compound N9 and moxifloxacin (MFX) had a synergistic effect as determined by the checkerboard assay in *M*. *tuberculosis* H37Rv.

Drug Combination	MIC (μg/mL)^a^		FICI^b^	Outcome
	Alone	Combined		
N9 INH	1.56 0.31	0.78 0.16	1.0	Indifferent
N9 INH	3.13 0.16	3.13 0.16	2.0	
N9 INH	3.13 0.31	1.56 0.16	1.0	
N9 RIF	1.56 0.02	0.78 0.005	0.75	
N9 RIF	3.13 0.15	1.56 0.009	0.56	
N9 RIF	3.13 0.15	1.56 0.04	0.75	
N9 EMB	1.56 1.25	0.78 0.63	1	
N9 EMB	3.13 5	3.13 5	2	
N9 EMB	3.13 5	3.13 5	2	
N9 MFX	1.56 0.15	0.20 0.04	0.38	Synergism
N9 MFX	3.13 0.15	0.10 0.04	0.28	
N9 MFX	3.13 0.15	0.40 0.04	0.38	

^*a*^Three independent experiments were performed.

^*b*^Values of FICI below 0.5 suggest a synergistic effect, between 0.5 and 4.0 indicate that each drug acts independently (indifferent), and above 4.0 suggest an antagonistic interaction [15]. INH, isoniazid; RIF, rifampicin; EMB, ethambutol; MFX, moxifloxacin

### Safety toxicology and metabolic interactions *in silico* study

Computational analysis of the compounds’ interactions with five isoforms of CYP450 reveals that all molecules are either substrates or inhibitors of at least one isoform. According to the pkCSM predictions, all compounds exhibited features of CYP1A2 and CYP2C19 inhibitors. N5 is the only compound that does not serve as substrate to CYP3A4, which could indicate a different metabolic route. Compounds N15, N17, and N36 can exhibit inhibitory effects against CYP2C9 and CYP3A4, thus compromising drug metabolism. It is possible that the occurrence of three methoxy groups at different positions of the six-membered rings of these compounds is involved in the inhibition mechanism. **S4 Table** summarized the predictions of the compounds interactions with CYP450 isoforms.

The analysis of toxicity effects revealed that the majority of compounds do not present any undesirable features, except N3 and N7. These two compounds have high risk to present mutagenic effects, and N7 has high risk to present adverse reproductive effects. The occurrence of a nitrogen dioxide group at the six-membered ring 4-position (N7) and the 1,3 benzodioxole moiety (N3) may be involved in these risks. **S5 Table** summarizes the predictions related to toxicity effects.

### Direct inhibition of CYP450 enzymes by N9

The inhibitory potential of N9 over the activity of the major CYP450 isoforms was experimentally evaluated by monitoring selective reactions catalyzed by those enzymes in the presence and the absence of different concentrations of N9, and further estimating the IC_50_. Results showed that N9 did not inhibit the activity of CYP2C19, CYP2D6, and CYP2E1 over the concentration range evaluated (0.01–100 μmol L^-1^), and the percentage of remaining enzyme activity maintained approximately 100%. For CYP3A4/5, two reactions were monitored as recommended by FDA guideline [25], nifedipine oxidation and midazolam 1’-hydroxylation. N9 did not inhibit the nifedipine oxidation (100% of remaining enzyme activity at 100 μmol L^-1^). Conversely, N9 presented a weak inhibitory potential (75% of remaining enzyme activity at 100 μmol L^-1^, IC_50_> 100 μmol L^-1^) as well as observed for CYP2C9 [26]. However, when CYP1A2 was evaluated, N9 exhibited a strong inhibitory potential, with an IC_50_ value of 2 μmol L^-1^ (**S2 Fig**) [26]. This observation was in accordance with *in silico* prediction assays.

CYP450 enzymes are responsible for the biotransformation of many drugs; inhibition of these enzymes can cause drug-drug interactions and adverse effects. CYP1A2, inhibited by N9, is one of the major CYP450 enzymes in the human liver (4–16% of the total content) [27] and plays an important role in the metabolism of clinically used drugs, contributing to about 9% of the drug metabolism pathways, such as the metabolism of caffeine, aldosterone, theophylline, clozapine, and tizanidine [28]. Moreover, N9 did not inhibit or weakly inhibited three important isoforms, CYP3A4, CYP2C9, and CYP2C19, which account for most of cytochrome metabolic system [29]. Future pharmacokinetic studies will elucidate if maximum plasma levels of N9 reach CYP1A2-inhibitory concentrations *in vivo*, and if this *in vitro* inhibition of CYP1A2 by this new chemical entity is clinically relevant. Taken together, these data are important to predict *in vivo* drug interaction and to guide further investigations.

### Toxicity evaluation

In the present study, chalcone N9 was not mutagenic to the five *S*. *typhimurium* strains used in the presence and absence of metabolic activation (**Table 3**): TA1535 and the corresponding isogenic strain TA100 which detect base pair substitutions (DNA target leucine [GAG] by proline [GGG]), TA98 (DNA target–C–G–C–G–C–G–C–G-; -1) and TA97a (DNA target -C-C-C-C-C-C-; +1 cytosine) which detect frameshift mutations, and TA102 which is sensitive to oxidative, crosslinking, and alkylating mutagens (transversions or transitions in TAA ochre). Additionally, we aimed at investigating the genotoxic potential of N9 by the alkaline comet assay. Interestingly, N9 tested to 10 μg/mL did not induce DNA damage in HepG2 cells, when compared to the controls (untreated group and DMSO-treated). MMS, a known mutagenic alkylating agent was used as positive control for DNA damage (**S3 Fig**).

**Table 3:** Induction of *his*^+^ revertants in *S*. *typhimurium* strains by chalcone N9 with and without metabolic activation (S9 mix).

*S*. *typhimurium* strains											
Substance	Concentration (μg/plate)	TA98		TA97a		TA100		TA1535		TA102	
		Rev/plate^a^	MI^b^	Rev/plate^a^	MI^b^	Rev/plate^a^	MI^b^	Rev/plate^a^	MI^b^	Rev/plate^a^	MI^b^
Without metabolic activation (-S9)											
NC^c^	-	34.3±4.4	-	127.3±10.5	-	115.3±18.2	-	11.3±2.3	-	337.7±44.7	-
N9	10	28.3±7.6	0.83	145.7±20.3	1.14	125.0±10.5	1.08	11.7±0.6	1.03	254.3±47.1	0.75
	50	25.0±6.1	0.73	125.0±12.5	0.98	101.0±12.5	0.88	9.3±0.6	0.82	299.3±50.6	0.89
	100	27.7±4.0	0.81	118.0±8.2	0.93	97.0±20.1	0.84	11.7±5.9	1.03	341.3±16.8	1.01
	200	30.5±2.1	0.89	124.3±13.3	0.98	109.7±7.8	0.95	13.3±4.0	1.18	324.3±4.2	0.96
	400	42.5±15.8	1.24	134.0±3.6	1.05	96.7±9.3	0.84	14.0±3.6	1.24	321.0±27.4	0.95
PC^d^	0.5 (4NQO) or 1 (NaN_3_)	169.3±19.9***	**4.93**	692.0±62.2***	**5.44**	695.5±40.3***	**6.03**	1133.0±179.6***	**100.0**	2020.0±141.4***	**5.98**
With metabolic activation (+S9)											
NC^c^	-	40.0±8.5	-	115.3±3.1	-	116.0±10.8	-	8.3±4.2	-	374.3±21.1	-
N9	10	43.7±4.7	1.09	128.0±12.1	1.11	108.7±15.2	0.94	9.7±1.2	1.16	376.3±29.0	1.01
	50	32.0±7.8	0.80	106.0±14.2	0.92	130.7±10.7	1.13	10.3±2.5	1.24	358.0±49.1	0.96
	100	37.7±5.7	0.94	113.5±5.0	0.98	112.7±15.5	0.97	8.3±4.9	1.00	368.7±12.7	0.99
	200	42.0±5.6	1.05	82.0±1.0	0.71	110.0±11.8	0.95	8.7±1.2	1.04	382.0±58.2	1.02
	400	42.3±6.7	1.06	104.0±28.8	0.90	112.0±13.9	0.97	5.7±1.2	0.68	371.0±23.6	0.99
PC^d^	5 (2-AA)	1586.0±161.2***	**39.65**	742.0±59.4***	**6.44**	1279.0±50.2***	**11.03**	58.0±12.7***	**6.96**	1354.0±21.1***	**3.62**

^a^Number of revertants/plate: mean of three independent experiments ± SD

^b^MI: mutagenic index (number of *his*^+^ induced in the sample/number of spontaneous *his*+ in the negative control)

^c^NC: negative control (10 μL DMSO: dimethylsulfoxide used as solvent for the chalcone).

^d^PC: positive control (-S9) sodium azide to TA100 and TA1535; 4-nitroquinoline-oxide to TA97a, TA98 and TA102; (+S9) 2-aminoanthracene; Significant differences compared to the negative control.

*** *P<*0.001.

## Conclusion

Despite the constant warnings of the World Health Organization, TB still presents high incidence and mortality worldwide. In addition, the current therapeutic protocols are not well-adhered to by patients, which increases the cases of resistant strains. This study revealed potent anti-tubercular activity for quinoxaline-derived chalcones. The lead compound N9 significantly inhibited growth of the *M*. *tuberculosis* H37Rv strain, and clinical isolates, at low concentrations (MICs between 1.56 μg/mL and 3.135 μg/mL). The anti-tubercular effects probably rely on mechanisms other than those used by INH, as chalcone N9 did not affect the synthesis of mycolic acids or non-hydroxylated fatty acids. In combination assays with clinically available drugs, the chalcone N9 demonstrated a synergistic effect when combined with MFX. It is tempting to infer that this combination might be an interesting alternative for the treatment of multi-resistant TB. In addition, the chalcone N9 did not display any mutagenic or genotoxic effects. *In silico* studies corroborated pharmacological safety of N9, since most chalcones did not show mutagenic, tumorigenic, irritant and reproductive effects. Despite N9 inhibited CYP1A2 isoform, it did not inhibit CYP3A4/5 and CYP2C19, and weakly inhibited CYP2C9 isoform.

In summary, N9 displayed a satisfactory efficacy against resistant *M*. *tuberculosis* strains, especially when combined with the quinolone MFX, with a favorable toxicity profile. Noteworthy, the mechanisms underlying the anti-tubercular effects of N9 are not likely related to pathways associated with resistance to INH or RIF. These results allow us to suggest that chalcone N9 is a potent lead molecule and might represent a candidate for clinical development as a novel anti-TB agent against resistant forms.

## Supporting information

S1 Table Selected reaction monitoring transition, optimized collision energy (CE), tube lens, and retention times for analysis of the metabolites formed from CYP450-mediated probe metabolism. (DOCX)

S2 Table Mass spectrometer conditions for analysis of the metabolites formed from CYP450-mediated probe metabolism. (DOCX)

S3 Table Incubation and extraction conditions for CYP450 inhibition assays *in vitro*. (DOCX)

S4 Table *In silico* prediction of the interaction between chalcones and cytochrome P450 isoforms. (DOCX)

S5 Table *In silico* prediction of the chacones’s toxicity effects. (DOCX)

S1 Fig Chromatographic analysis of *M*. *tuberculosis* lipids after incubation, for 18 hours, with the compound N9 at 10 and 25 μg/mL (lanes 3 and 4, respectively), using INH 0.5 μg/mL (lane 2) as positive control. Lane 1 represents the untreated control. Two independent experiments were performed, as previously described [Rodrigues-Junior VS, Junior AAS., Villela, AD, Belardinelli JM, Morbidoni HR, Basso LA, et al. IQG-607 abrogates the synthesis of mycolic acids and displays intracellular activity against *Mycobacterium tuberculosis* in infected macrophages. Int J Antimicrob Agents 2014;43:82–5].
(TIF)

S2 Fig Inhibitory potential of N9 on CYP1A2-catalyzed phenacetin O-deethylation using human liver microsomes (each point is reported as mean ± SD, n = 3). (PDF)

S3 Fig DNA damage index, measured by alkaline comet assay in HepG2 cells after 24 h of N9 (2.5, 5, or 10 μg/mL) or MMS (0.1 mM; positive control) exposure. NC represents the negative control groups while DMSO represents the group incubated with DMSO 0.5%, the vehicle used for the treatments. Data is represented by mean±SD. One-way ANOVA followed by Dunett's post-test were used in the statistical analyses; ***P<0.001.
(TIF)

## Abbreviations

CYP450: cytochrome P450
EMB: ethambutol
FICI: fractional inhibitory concentration index
HLMs: human liver microsomes
IC_50_: half-maximum inhibitory concentration
INH: isoniazid
IS: internal standard
MFX: moxifloxacin
MMS: methyl methanesulfonate
MIC: minimum inhibitory concentration
REMA: resazurin reduction microplate assay
RIF: rifampicin
SRM: selective reaction monitoring
TB: tuberculosis
