# Supplementary material for: Pre-clinical evaluation of quinoxaline-derived chalcones in tuberculosis
Source: PLoS One. 2018 Aug 16;13(8):e0202568. doi: 10.1371/journal.pone.0202568 (PMC6095594; doi:10.1371/journal.pone.0202568)
Supplement: S1 Table — (DOCX) [file pone.0202568.s001.docx]

| **Analyte** | **ESI** | **Precursor ion (Q1)** | **Quantification ion (CE)** | **Confirmation ion (CE)** | **Tube lens** | **Retention time (min)** |
| --- | --- | --- | --- | --- | --- | --- |
| acetaminophen | + | 151.9 | 110.0 (15) | 65.2 (31) | 71 | 1.82 |
| 4′-hydroxydiclofenac | + | 311.8 | 230.8 (18) | 265.8 (12) | 66 | 2.17 |
| 4′-hydroxymephenytoin | - | 232.9 | 190.0 (12) | 160.9 (19) | 93 | 1.82 |
| 1′-hydroxybufuralol | + | 277.9 | 185.9 (17) | 241.9 (13) | 97 | 1.46 |
| 6-hydroxychlorzoxazone | - | 183.8 | 119.8 (20) | 64.1 (30) | 103 | 1.91 |
| 1′-hydroxymidazolam | + | 342.0 | 323.9 (20) | 202.8 (26) | 91 | 2.01 |
| dehydronifedipine | + | 345.0 | 283.9 (27) | 267.8 (27) | 86 | 2.05 |
| diazepam | + | 284.9 | 192.9 (31) | 153.9 (26) | 100 | 2.05 |
| tolbutamide | - | 268.9 | 169.8 (20) | 105.9 (35) | 102 | 2.06 |
