# Supplementary material for: Pre-clinical evaluation of quinoxaline-derived chalcones in tuberculosis
Source: PLoS One. 2018 Aug 16;13(8):e0202568. doi: 10.1371/journal.pone.0202568 (PMC6095594; doi:10.1371/journal.pone.0202568)
Supplement: S2 Table — (DOCX) [file pone.0202568.s002.docx]

| **CYP isoform** | **Cone voltage (V)** | **Capillary temperature (ºC)** | **Vaporizer temperature (ºC)** | **Sheath gas*** | **Ion sweep gas*** | **Auxiliary gas*** |
| --- | --- | --- | --- | --- | --- | --- |
| CYP1A2 | 5000 | 280 | 340 | 30 | 0 | 5 |
| CYP2C9 | 3500 | 375 | 205 | 50 | 2 | 15 |
| CYP2C19 | 5000 | 375 | 200 | 60 | 2 | 35 |
| CYP2D6 | 4000 | 380 | 225 | 20 | 0 | 5 |
| CYP2E1 | 4000 | 280 | 200 | 40 | 2 | 5 |
| CYP3A4/5^a^ | 4500 | 180 | 200 | 10 | 0 | 5 |
| CYP3A4/5^b^ | 4000 | 400 | 260 | 30 | 2 | 15 |

(^*^) Arbitrary units; (^a^) Midazolam 1’-hydroxylation reaction; (^b^) Nifedipine oxidation reaction
