# Supplementary material for: Pre-clinical evaluation of quinoxaline-derived chalcones in tuberculosis
Source: PLoS One. 2018 Aug 16;13(8):e0202568. doi: 10.1371/journal.pone.0202568 (PMC6095594; doi:10.1371/journal.pone.0202568)
Supplement: S3 Table — (DOCX) [file pone.0202568.s003.docx]

| **CYP**  **isoform** | **Probe**  **substrate** | **Metabolite** | **Substrate concentration**  **(μmol L^-1^)** | **HLM**  **(mg mL^-1^)** | **Incubation**  **time (min)** | **Extractor**  **solvent** | **Modifier** | **Internal**  **standard** |
| --- | --- | --- | --- | --- | --- | --- | --- | --- |
| CYP1A2 | Phenacetin | Acetaminophen | 12.03 | 0.30 | 30 | Ethyl acetate | - | Diazepam |
| CYP2C9 | Diclofenac | 4′-Hydroxydiclofenac | 49.45 | 0.10 | 20 | Chloroform | 100 µL HCl  1 mol L^-1^ | Diazepam |
| CYP2C19 | S-Mephenytoin | 4′-Hydroxymephenytoin | 44.83 | 0.20 | 60 | Ethyl acetate | - | Diazepam |
| CYP2D6 | Bufuralol | 1′-Hydroxybufuralol | 5.40 | 0.25 | 30 | Ethyl acetate | 100 µL NaOH  2.5 mol L^-1^ | Diazepam |
| CYP2E1 | Chlorzoxazone | 6-Hydroxychlorzoxazone | 145.90 | 0.40 | 20 | Chloroform | - | Tolbutamide |
| CYP3A4/5 | Midazolam | 1′-Hydroxymidazolam | 5.41 | 0.10 | 10 | Ethyl acetate | 100 μL NaOH  1 mol L^-1^ | Diazepam |
| CYP3A4/5 | Nifedipine | Dehydronifedipine | 7.00 | 0.15 | 15 | Ethyl acetate | - | Diazepam |

|  |  |  |  |  |
| --- | --- | --- | --- | --- |
