# Supplementary material for: Pre-clinical evaluation of quinoxaline-derived chalcones in tuberculosis
Source: PLoS One. 2018 Aug 16;13(8):e0202568. doi: 10.1371/journal.pone.0202568 (PMC6095594; doi:10.1371/journal.pone.0202568)
Supplement: S4 Table — (DOCX) [file pone.0202568.s004.docx]

|  |  |  |  |  |  |  |  |
| --- | --- | --- | --- | --- | --- | --- | --- |
| **Compound** | **Substrate** | | **Inhibitor** | | | | |
|  | **CYP2D6** | **CYP3A4** | **CYP1A2** | **CYP2C19** | **CYP2C9** | **CYP2D6** | **CYP3A4** |
| **N3** | - | + | + | + | - | - | - |
| **N4** | - | + | + | + | + | - | - |
| **N5** | - | - | + | + | - | - | - |
| **N7** | - | + | + | + | - | - | - |
| **N9** | - | + | + | + | - | - | - |
| **N10** | - | + | + | + | - | - | - |
| **N15** | - | + | + | + | + | - | + |
| **N16** | - | + | + | + | - | - | - |
| **N17** | - | + | + | + | + | - | + |
| **N19** | - | + | + | + | - | - | - |
| **N20** | - | + | + | + | - | - | - |
| **N23** | - | + | + | + | - | - | - |
| **N33** | - | + | + | + | - | - | - |
| **N34** | - | + | + | + | - | - | - |
| **N36** | - | + | + | + | + | - | + |
|  |  |  |  |  |  |  |  |
