# Supplementary figures and images for: Pre-clinical evaluation of quinoxaline-derived chalcones in tuberculosis
Source: PLoS One. 2018 Aug 16;13(8):e0202568. doi: 10.1371/journal.pone.0202568 (PMC6095594; doi:10.1371/journal.pone.0202568)

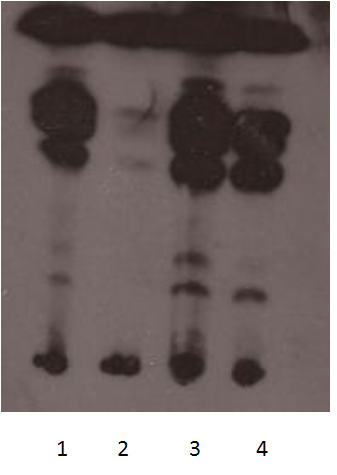

Supplement: S1 Fig — Lane 1 represents the untreated control. Two independent experiments were performed, as previously described [Rodrigues-Junior VS, Junior AAS., Villela, AD, Belardinelli JM, Morbidoni HR, Basso LA, et al. IQG-607 abrogates the synthesis of mycolic acids and displays intracellular activity against Mycobacterium tuberculosis in infected macrophages. Int J Antimicrob Agents 2014;43:82–5]. (TIF) [file pone.0202568.s006.TIF]

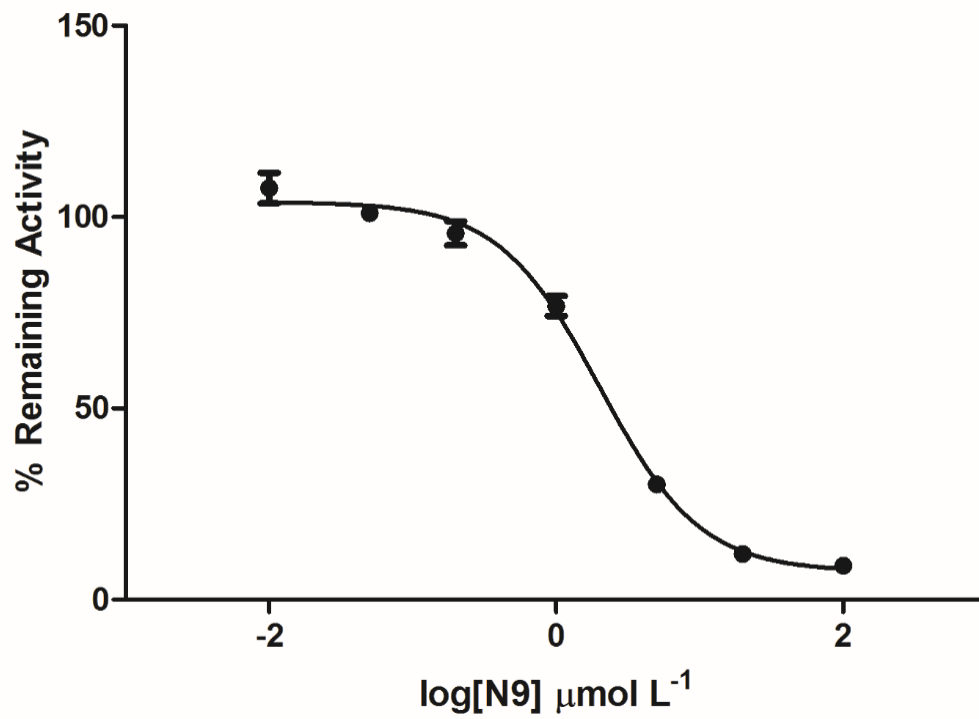

Supplement: S2 Fig — (PDF) [file pone.0202568.s007.pdf]

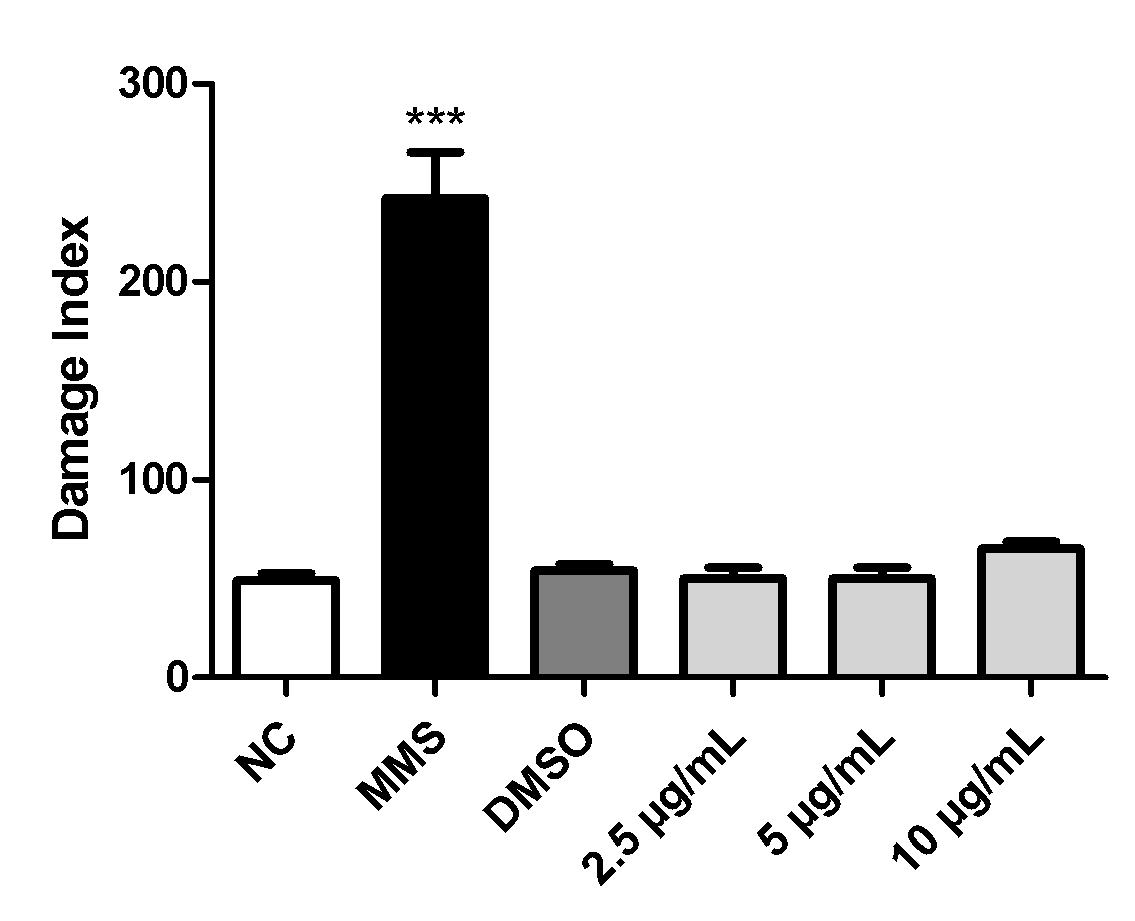

Supplement: S3 Fig — NC represents the negative control groups while DMSO represents the group incubated with DMSO 0.5%, the vehicle used for the treatments. Data is represented by mean±SD. One-way ANOVA followed by Dunett's post-test were used in the statistical analyses; ***P<0.001. (TIF) [file pone.0202568.s008.tif]
